# Bringing optimised COVID-19 vaccine schedules to immunocompromised populations (BOOST-IC): study protocol for an adaptive randomised controlled clinical trial

**DOI:** 10.1186/s13063-024-08315-2

**Published:** 2024-07-17

**Authors:** David W. J. Griffin, Michael Dymock, Germaine Wong, C. Orla Morrissey, Sharon R. Lewin, Allen C. Cheng, Kirsten Howard, Julie A. Marsh, Kanta Subbarao, Michelle Hagenauer, Janine Roney, Anthony Cunningham, Tom Snelling, James H. McMahon

**Affiliations:** 1 Department of Infectious Diseases, Alfred Hospital and School of Translational Medicine, Monash University, Melbourne, Australia; 2 Wesfarmers Centre of Vaccines and Infectious Diseases, Telethon Kids Institute, Perth, Australia; 3 Centre for Kidney Research, The Children’s Hospital at Westmead, Westmead, NSW Australia; 4 Department of Renal Medicine, Westmead Hospital, Westmead, NSW Australia; 5 Sydney School of Public Health, Faculty of Medicine & Health, University of Sydney, Sydney, NSW Australia; 6 Department of Infectious Diseases, The University of Melbourne at the Peter Doherty Institute for Infection and Immunity, Melbourne, Australia; 7 Victorian Infectious Diseases Service, Royal Melbourne Hospital, at the Peter Doherty Institute for Infection and Immunity, Melbourne, Australia; 8 Department of Infectious Diseases, Monash Medical Centre, Melbourne, Australia; 9 Monash University School of Clinical Sciences at Monash Health, Clayton, Australia; 10 Menzies Centre for Health Policy and Economics, Faculty of Medicine and Health, University of Sydney, Sydney, NSW Australia; 11 Centre for Child Health Research, School of Medicine, The University of Western Australia, Perth, Australia; 12 WHO Collaborating Centre for Reference and Research On Influenza at the Peter Doherty Institute for Infection and Immunity, Melbourne, Australia; 13 Department of Microbiology and Immunology, University of Melbourne at the Peter Doherty Institute for Infection and Immunity, Melbourne, Australia; 14 Westmead Institute for Medical Research, University of Sydney, Sydney, NSW Australia

**Keywords:** COVID-19, mRNA vaccine, Immunisation, HIV, Solid organ transplantation, Chronic lymphocytic leukaemia, Non-Hodgkin lymphoma, Multiple myeloma, RCT

## Abstract

**Background:**

Immunocompromised hosts (ICH) experience more breakthrough infections and worse clinical outcomes following infection with COVID-19 than immunocompetent people. Prophylactic monoclonal antibody therapies can be challenging to access, and escape variants emerge rapidly. Immunity conferred through vaccination remains a central prevention strategy for COVID-19. COVID-19 vaccines do not elicit optimal immunity in ICH but boosting, through additional doses of vaccine improves humoral and cellular immune responses. This trial aims to assess the immunogenicity and safety of different COVID-19 vaccine booster strategies against SARS-CoV-2 for ICH in Australia.

**Methods:**

Bringing optimised COVID-19 vaccine schedules to immunocompromised populations (BOOST-IC) is an adaptive randomised trial of one or two additional doses of COVID-19 vaccines 3 months apart in people living with HIV, solid organ transplant (SOT) recipients, or those who have haematological malignancies (chronic lymphocytic leukaemia, non-Hodgkin lymphoma or multiple myeloma). Key eligibility criteria include having received 3 to 7 doses of Australian Therapeutic Goods Administration (TGA)-approved COVID-19 vaccines at least 3 months earlier, and having not received SARS-CoV-2-specific monoclonal antibodies in the 3 months prior to receiving the study vaccine. The primary outcome is the geometric mean concentration of anti-spike SARS-CoV-2 immunoglobulin G (IgG) 28 days after the final dose of the study vaccine. Key secondary outcomes include anti-spike SARS-CoV-2 IgG titres and the proportion of people seroconverting 6 and 12 months after study vaccines, local and systemic reactions in the 7 days after vaccination, adverse events of special interest, COVID-19 infection, mortality and quality of life.

**Discussion:**

This study will enhance the understanding of COVID-19 vaccine responses in ICH, and enable the development of safe, and optimised vaccine schedules in people with HIV, SOT, or haematological malignancy.

**Trial registration:**

ClinicalTrials.gov NCT05556720. Registered on 23rd August 2022.

**Supplementary Information:**

The online version contains supplementary material available at 10.1186/s13063-024-08315-2.

## Introduction

### Background and rationale {6a}

The COVID-19 pandemic has had a disproportionate impact on the health, wellbeing, and care of immunocompromised hosts (ICH) [1]. Early evidence demonstrated that people living with HIV (PLWH), those with solid organ transplants (SOT) and patients with haematological malignancies are at least twice as likely to experience COVID-19-related hospitalisation, intensive care admission and death compared to the age and sex-matched population [2–5]. Hence, strategies to reduce the incidence of COVID-19 infection and prevent the associated complications in these groups, including vaccination, remain central to public health efforts [6].

The higher likelihood of complications experienced by ICH may be dependent on co-existing comorbidities and level of immunosuppression as both innate and adaptive immunity is required to control the viral replication. PLWH, who experience earlier onset and a higher burden of age-associated comorbidities compared with the general population [7], have been shown to have a small, but potentially clinically important reduction in seroconversion and neutralisation responses following COVID-19 vaccination [6]. Those with low CD4 counts were less likely to demonstrate seroconversion or neutralisation following a primary course of COVID-19 vaccination [6, 8]. Similarly, several clinical trials and observational studies have shown significantly reduced humoral responses to standard two-dose schedules of COVID-19 vaccines, and a higher incidence of breakthrough COVID-19 infections in SOT recipients [9–11]. Several factors have been associated with decreased COVID-19 immune responses in SOT recipients, including the net state of immunosuppression and time from transplantation; the immunosuppressive regimen, including the administration of mycophenolate mofetil and prior use of T cell depleting agents as induction therapies [12]; and previous infection with COVID-19 and other coronaviruses [13–16]. While people with haematological malignancies are a heterogenous group, they experience some of the poorest responses to vaccination [17]. The type of haematological malignancy and type and timing of chemotherapy relative to vaccination are key predictors of attenuated immunity, with B-lymphocyte depleting therapies, like anti-CD20 monoclonal antibodies and Bruton’s tyrosine kinase inhibitors having deleterious effects on humoral immunity [17–19].

International guidelines recommend additional doses of COVID-19 vaccines for ICH [19, 20] though recommendations vary regarding the number and frequency of booster doses. Although a growing body of evidence illustrates that additional doses of COVID-19 vaccines enhance humoral responses among ICH [21–23], but the optimal boosting strategies, including the number of doses and type of vaccine, remain unclear [24–26].

High-quality, randomised controlled clinical trials of different boosting strategies are therefore needed to optimise COVID-19 vaccination in ICH, to enhance protective immunity, while maintaining an acceptable safety profile.

### Objectives {7}

This study aims to assess humoral and cellular responses and confirm the safety of an additional one or two doses of an approved COVID-19 vaccine, 3 months apart, in PLWH, SOT recipients, and those with haematological malignancy. Specifically, we aim to examine how additional doses of COVID-19 vaccine/s affect the geometric mean concentration of anti-spike SARS-CoV-2 IgG antibody, 28 days after completion of the study vaccine, and other correlates of protective immunity.

### Trial design {8}

BOOST-IC is a multicentre, adaptive platform randomised trial, designed to compare up to three different vaccines at a given time. The design allows for the addition or removal of existing arms, in response to changes in recommendations from Australian authorities. This protocol is reported in keeping with the SPIRIT guideline [27].

## Methods: participants, interventions, and outcomes

### Study setting {9}

Eligible participants will be recruited in the outpatient setting at up to 16 academic hospitals in Australia (Appendix).

### Eligibility criteria {10}

Inclusion criteria

- Able to give informed consent and undertake study procedures
- Age ≥ 16 years old
- Have completed 3 to 7 doses of Australian Therapeutic Goods Administration (TGA)-approved SARS-CoV-2 vaccine (including mRNA [Pfizer or Moderna], ChAdOx1 [Oxford/Astra Zeneca] or protein [Novavax]) at least 3 months prior
- Fit the criteria to be included in one of the three following ICH groups:
  - ο Infected with HIV
  - ο Recipient of a SOT including kidney, pancreas, liver, heart, lung, or combination of these organs at least 6 weeks prior and without episodes of severe rejection requiring T- or B-cell depleting agents in the prior 3 months
  - ο Undergoing chemotherapy, immunotherapy and/or targeted therapy, or completed in the last 2 years for chronic lymphocytic leukaemia (CLL), non-Hodgkin lymphoma (NHL), or multiple myeloma (MM).

Exclusion Criteria

- Contraindicated to receive a COVID-19 booster vaccine (e.g. history of anaphylaxis to a vaccine component or myocarditis attributed to previous receipt of an mRNA vaccine).
- Has had less than 3 or more than 7 doses of COVID-19 vaccine
- Is on another clinical trial investigating alternate COVID-19 vaccination schedules or investigational drugs to prevent or treat COVID-19
- Life expectancy < 12 months or enrolment deemed not in the best interest of the patient
- Unable to provide informed consent
- Receipt of SARS-CoV-2 specific monoclonal antibodies in the 3 months prior to receiving the first dose of study vaccine
- Acute respiratory tract infection and/or temperature > 38 degrees on the day of receipt of the first dose of the study vaccine
- History of autologous stem cell transplant in the prior 6 months or history of ever having an allogeneic stem cell transplant or CAR T-cell therapy
- Have not received another licensed vaccine in the 7 days before or plan to receive one in the 7 days after the day of receiving the COVID-19 study vaccine (note: participants can receive another licensed vaccine on the same day as the COVID-19 vaccine)

### Who will take informed consent? {26a}

Patients will only be included after providing written informed consent. Consent will be obtained by a member of the research team, who will ensure the participant is fully informed of the nature and objectives of the research, its voluntary nature, and any possible risks of participation, prior to data collection or the commencement of any study-related procedures. Where appropriate, accredited interpreters will be used to ensure the consent process occurs in the participant’s preferred language.

### Additional consent provisions for collection and use of participant data and biological specimens {26b}

All participants will provide consent to use deidentified data and blood specimens to determine the primary and secondary outcomes of the study. In addition, a subset of individuals can consent to provide additional blood samples for use in future research.

## Interventions

### Explanation for the choice of comparators {6b}

Vaccines for the prevention of COVID-19 infection and severe disease approved by the Australian TGA can be included in this trial. Candidate vaccines include mRNA and protein subunit vaccines. The choice of which vaccines to include in the platform is done by the trial steering committee and can change over time. Decisions on which vaccines to include in the trial are based on: availability, the current clinical environment including variants of concern, scientific merit and contemporary recommendations from the Australian Technical Advisory Group on Immunisation (ATAGI). This study does not include a placebo arm.

### Intervention description {11a}

At any one time, a minimum of 1 and a maximum of 3 vaccine candidates can be under investigation. At the time of writing, 2 vaccines are currently being studied in the trial. These are monovalent mRNA vaccines encoding XBB.1.5 Omicron spike proteins, manufactured by Pfizer and Moderna, and currently recommended as COVID-19 booster vaccines. These are associated with higher neutralising antibody titres to contemporary variants and have a comparable safety profile when compared with ancestral vaccines [28–37].

The vaccines used in this study have evolved in accordance with contemporary recommendations from ATAGI, and local vaccine availability. All randomised participants in receipt of current or past study vaccines will be included in the final analyses.

Vaccines previously used for participants enrolled in the trial but which are no longer included as a study intervention are:

- mRNA ancestral/Omicron BA.1 Bivalent (Pfizer)
- mRNA ancestral/Omicron BA.1 Bivalent (Moderna)
- mRNA ancestral/Omicron BA.4/5 Bivalent (Pfizer)
- mRNA ancestral/Omicron BA.4/5 Bivalent (Moderna)

All participants will receive either one or two doses of COVID-19 vaccine, prepared, stored and administered from multidose vials in accordance with the manufacturers’ instructions.

### Criteria for discontinuing or modifying allocated interventions {11b}

Participants may discontinue participation or withdraw their consent without penalty. An investigator may also discontinue a participant from trial intervention if the participant demonstrates significant non-compliance with the trial intervention; experiences a serious or intolerable adverse event such that continued trial intervention would not be in the best interest of the participant; or requires early discontinuation for any other reason.

### Strategies to improve adherence to interventions {11c}

Site investigators and trial coordinators will monitor the completion of safety questionnaires and contact participants if these are incomplete to see if any additional information or assistance is required to complete these tasks. Study site coordinators are educated to encourage attendance and contact participants missing any follow-up visits to maximise completeness of the study data. In addition, the study schedule allows for enrolment and the initial study vaccine on the same day to minimise study visits.

### Relevant concomitant care permitted or prohibited during the trial {11d}

Participants should receive standard care throughout the study period. Participants may receive another licensed vaccine (e.g. influenza vaccine) in the opposite upper limb, concurrent with their trial vaccine. However, the other licensed vaccine cannot be administered in the period 7 days before or 7 days after the COVID-19 vaccine, to avoid misattribution of reactogenicity to the study vaccine.

### Provisions for post-trial care {30}

Post-trial care is the responsibility of the treating clinicians; Ancillary and post-trial care are not planned. If a participant experiences an adverse event following immunisation after the trial, they should seek medical review from their primary care clinician or the emergency department, as appropriate. Noting that trial follow-up continues for 12 months after COVID-19 vaccines have been completed.

### Outcomes {12}

The primary outcome is defined as the geometric mean concentration (GMC) of anti-spike IgG antibody against SARS-CoV-2, 28 days after completion of trial vaccine/s, i.e. after one or two doses in the respective trial arms.

Secondary outcomes include laboratory, safety and clinical outcomes:

Laboratory outcomes

- The GMC of anti-spike IgG antibody against SARS-CoV-2, 6 and 12 months after completion of trial vaccine/s
- The proportion of participants seropositive to SARS-CoV-2 IgG, 1, 6 and 12 months after completion of trial vaccine/s
- The GMC of anti-spike IgG antibody targeting specific SARS-CoV-2 variants, 1, 6 and 12 months after completion of trial vaccine/s
- The SARS-CoV-2 neutralising antibody response, 1, 6 and 12 months post completion of trial vaccine/s, in a subset of participants with response defined as either rise in the neutralising antibody titre
  - ο Fourfold rise in the neutralising antibody titre for those with detectable neutralising antibodies at baseline, OR
  - ο Detectable neutralisation in those with no detectable neutralising antibodies at baseline
- The magnitude of SARS-CoV-2 specific T-cell responses, 1, 6 and 12 months post completion of trial vaccine/s in a subset of participants
- Subset analysis and polyfunctionality (number of effector cytokines) of SARS-CoV-2 specific T-cell responses, 1, 6 and 12 months post completion of trial vaccine/s in a subset of participants

Safety outcomes

- Local and systemic reactions assessed on days 1, 2, 3, 4, 5, 6 and 7 after randomisation.
- Hospitalisation resulting from adverse events following immunisation (AEFI) up to day 28.
- Adverse events of special interest (AESI) up to 12 months post completion of trial vaccine/s

Clinical outcomes

- History of PCR-confirmed OR rapid antigen test (RAT) positive SARS-CoV-2 infection in participants up to 12 months post completion of trial vaccine/s
- PCR-confirmed OR rapid antigen test (RAT) positive SARS-CoV-2 infection requiring attendance at a medical facility for assessment and/or hospital admission up to 12 months post completion of trial vaccine/s
- Mortality due to any cause up to 12 months post completion of trial intervention
- Mortality due to SARS-CoV-2 infection up to 12 months post completion of trial intervention
- Need for oxygen therapy and/or ventilatory support due to SARS-CoV-2 infection up to 12 months post completion of trial vaccine/s
- Need for ICU care due to SARS-CoV-2 infection up to 12 months post completion of trial vaccine/s
- Quality of life estimates (EQ-5D-5L) at 1, 6 and 12 months post completion of trial vaccine/s
- Healthcare utilisation including outpatient pharmaceutical and medical service use and inpatient hospital admissions related to COVID-19 and study vaccines.

### Participant timeline {13}

**Fig. 1:**
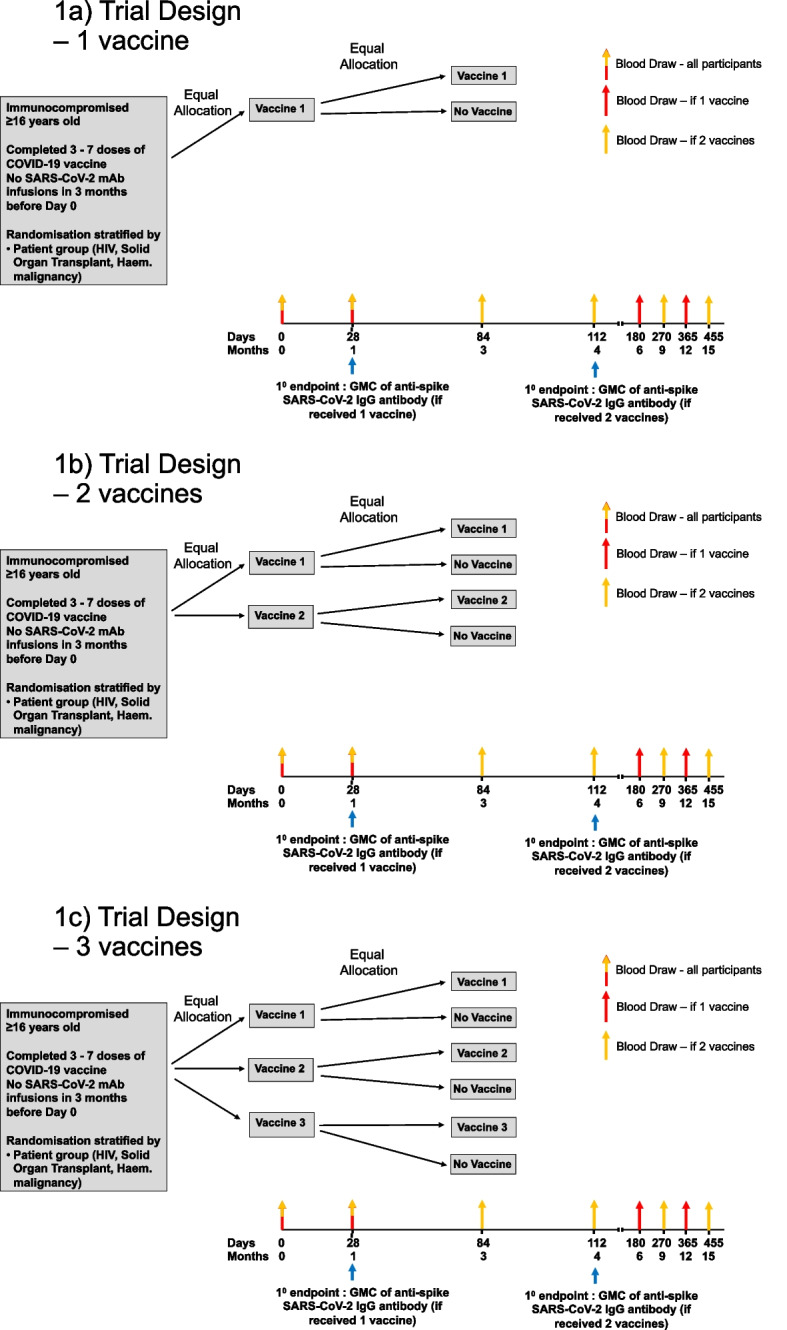
Trial design if **a**) one COVID-19 vaccine is included in the trial, **b**) if two COVID-19 vaccines are included in the trial, and **c**) if three COVID-19 vaccines are included in the trial

The study design and participant timeline are detailed in Fig. 1 and encompass a minimum of 1 and a maximum of 3 different study vaccines. Table 1 details the schedule of activities. Participants will have up to 7 visits during the study period, with follow-up continuing for 12 months following their final study vaccine. Participants randomised to receive one study vaccine will have 5 study visits, while those randomised to two doses of vaccine will have 7 visits. Participants following assigned study procedures can remain on the study for 13 to 16 months.

**Table 1:** Schedule of activities

Protocol activities										
	**Visit 1**^**c**^ **Screening**	**Visit 2**^**c**^ **Baseline**	**Visit 3**	**Visit 4 **^**a**^	**Visit 5**^**a**^	**Visit 6**^**b**^	**Visit 7**^**a**^	**Visit 8**^**b**^	**Visit 9**^**a**^	**Unscheduled visit **^**f**^
Visit window	Up to 4 weeks pre M0	M0	M1 (D28) + / − 7 days	M3 (D84) + / − 7 days	M4 (D112) + / − 7 days	M6 (D180) + / − 30 days	M9 (D 270) + / − 30 days	M12 (D 365) + / − 30 days	M15 (D455) + / − 30 days	
Informed consent	✓									
Medical history, incl/excl criteria	✓									
Symptoms, adverse events,		✓	✓	✓	✓	✓	✓	✓	✓	✓
QOL		✓	✓		✓	✓	✓	✓	✓	✓
Vaccine administered		✓		✓						
SARS-CoV-2 IgG assessment		✓^**d**^	✓^**d**^	✓^**d**^	✓^**d**^	✓^**d**^	✓^**d**^	✓^**d**^	✓^**d**^	✓^**d**^
Blood to process and store as plasma and PBMCs^e^		✓	✓	✓	✓	✓	✓	✓	✓	✓

Notes: *M* month, *QOL* quality of life, *PBMCs* peripheral blood mononuclear cells

^a^ If randomised to a second study vaccine

^b^ If only receiving one study vaccine

^c^ Screening and baseline procedures can be completed on the same day

^d^ Quantitative ELISA for SARS-CoV-2 anti-spike IgG

^e^ Immunogenicity subset including SARS-CoV-2 neutralisation assay (see section below)

^f^ An example of an unscheduled visit is for individuals receiving anti-SARS-CoV-2 monoclonal antibodies on study and attending for additional assessment of SARS-CoV-2 IgG, ideally in the 3 days before and 3 days after the infusions to assess antibody kinetics

After receipt of each vaccine, participants will be asked to complete a survey on a secure web-based portal regarding their experience of local and systemic reactions following the trial intervention, daily for 7 days.

### Sample size {14}

The maximum sample size is 960 participants, including 320 in each of the HIV, SOT, and haematological malignancy groups. This maximum sample size is based on simulations conducted prior to trial commencement for estimating the primary outcome (anti-spike SARS-CoV-2 IgG antibodies 28 days after completion of trial vaccine/s) for different trial strata (clinical group [HIV, SOT, haematological malignancy], vaccine brand, SARS-CoV-2 serostatus at enrolment) with sufficient precision. The simulations are detailed in the Statistical Appendix and will be published separately.

### Recruitment {15}

Research staff at participating sites will be trained on trial requirements to ensure eligible participants are identified, provided with appropriate information, and invited to participate in the trial. Eligible participants are identified across participating sites by clinicians involved in the care of eligible patient groups in the inpatient and ambulatory setting. Recruitment strategies will use approved material to target the study population and specific subgroups of participants based on medical and COVID-19 vaccination history.

## Assignment of interventions: allocation

### Sequence generation {16a}

Participants will be assigned to an arm using a central computer-generated random allocation algorithm. Randomisation will be stratified by study subgroup (HIV, SOT, haematological malignancy).

Participants will be equally randomised to one of the available vaccine arms if an intervention arm is dropped after an interim analysis or following a recommendation from the data safety management committee, then future participants will be equally allocated to the remaining arms.

### Concealment mechanism {16b}

After central randomisation, individual assignments will be delivered securely and confidentially to the identified unblinded site personnel administering the vaccines via a web-based portal and dispensed from an onsite clinical trials pharmacy by an unblinded pharmacist. At vaccination, an un-blinded member of the research team will dispense and administer the study vaccine to a blinded participant. Pre-filled syringes containing an allocated approved COVID-19 booster vaccine will be covered with opaque tape and concealed in an opaque box until ready for administration. Prior to opening the box, the participant will be asked to look away. The member of the research team will administer the trial vaccine per Australian guidelines.

### Implementation {16c}

Participants will be enrolled by the research team and will be randomly assigned to interventions as described, using an allocation sequence generated by study statisticians. Trial vaccines will be administered as described, to ensure allocation concealment.

## Assignment of interventions: blinding

### Who will be blinded {17a}

Participants and clinicians involved in their care will be blinded to intervention allocation, though will become unblinded to the administration of a second study vaccine dose after the 1-month visit. This is because immunisations administered to participants over the duration of the study need to be recorded on the Australian Immunisation Register (AIR) by the un-blinded vaccine nurse. The type and date of the vaccine administered will be recorded in the study database and uploaded to AIR 6 weeks following randomisation. As participants are then able to look up their study vaccine on the AIR, they have the potential to become unblinded from this point onwards.

Research staff involved in the follow-up of adverse reactions will be blinded to the group assignment of the participants. Laboratory staff processing or analysing trial specimens will be blinded to the group assignment of participants.

Trial Pharmacists, preparing interventions, research staff administering vaccine doses, and a nominated trial statistician who will prepare interim reports for the data safety and monitoring committee will be unblinded.

### Procedure for unblinding if needed {17b}

In the event of compelling medical, safety or other concerns, a central password-protected randomisation list can be accessed by the trial statistician, upon authorisation by the principal investigator or their delegate, during business hours. Participants for whom the assignment is revealed will remain in the study and where possible continue to receive surveys and attend scheduled study visits.

## Data collection and management

### Plans for assessment and collection of outcomes {18a}

After enrollment, research nurses will collect relevant demographic (age, sex and ethnicity) and clinical data (comorbidities, immunosuppression, and COVID-19 vaccination and infection), in accordance with a trial-specific case report form (CRF) at the baseline visit. Participants are scheduled for either 5 or 7 trial visits (Table 1), depending on whether randomised to one or two vaccine booster doses. From day 1 to 7 following study vaccine administration, participants will be asked to complete a daily electronic vaccine diary card delivered via email or text message.

Table 1 outlines the study schedule. Blood samples will be collected at baseline and each follow-up visit, for assessment of immunological outcomes. At each study visit, a research nurse will collect relevant clinical data per the CRF, review incomplete diary card entries as appropriate, and assess for serious adverse events since the prior study visit. Quality of life assessment will be conducted using the validated EQ-5D-5L tool [38].

### Plans to promote participant retention and complete follow-up {18b}

Data will be entered at each visit, by a study nurse, and checked periodically for completion by the primary study coordinator. Participant vaccine diary cards are checked for completion and can be completed by a study nurse by telephone if needed. Participants will be contacted to schedule follow-up visits in the event of non-attendance.

### Data management {19}

All clinical trial data will be stored in a password-protected, encrypted, study-specific, online clinical trial management system (CTMS) hosted by the sponsor, Monash University. The CTMS includes the electronic CRF, adverse event, EQ-5D-5L and protocol deviation forms. A paper version of the CRF will be available where a digital version is not. Data will be entered into the CTMS by trained study nurses. Participant-reported data from electronic diary cards will populate a linked patient-reported measurement system (PRMS). Data will be stored indefinitely, backed up regularly, and only accessible by researchers involved in this study. Regular data quality checks will be performed to identify data that appear inconsistent, incomplete, or inaccurate, by trained study coordinators.

### Confidentiality {27}

Participant confidentiality will be strictly maintained by the participating investigators and research staff. Participants will be allocated a participant identification number at enrolment, with personal identifiers maintained in a separate database. This confidentiality will be extended to cover testing of biological samples and genetic tests in addition to the clinical information relating to participants. Password protection and user right management will be used for the CTMS, ensuring only authorized study personnel have access to the data during and after the study.

The trial protocol, documentation, data, and all other information generated will be held in confidence. No information concerning the study, or the data will be released to any unauthorised third party, without prior written approval of the sponsoring institution. No personal identifiers will be reported in publications or presentations.

### Plans for collection, laboratory evaluation and storage of biological specimens for genetic or molecular analysis in this trial/future use {33}

Stored samples may be used for future analyses related to COVID-19 research, including genetic testing and HLA-typing, conditional to adherence to local regulations and ethical guidelines. Genetic testing will be for research purposes and not suitable for clinical purposes and therefore the results, regardless of the findings, will not be returned to the participant.

All future collaborations using biological or other specimens would require approval by the steering committee and need to obtain relevant ethics approval and materials transfer agreement. If any laboratory specimens or other records leave a study site, they will be identified only by the Participant Identification Number to maintain confidentiality.

## Statistical methods

### Statistical methods for primary and secondary outcomes {20a}

Baseline demographic and anthropometric data as well as the defined safety and immunogenicity outcome measures will be stratified by study cohort (HIV, SOT, haematological malignancy) and summarised with appropriate measures of spread. Immune responses and reactogenicity to COVID-19 vaccines will be assessed on a modified intention-to-treat population, depending on actual intervention received between randomisation and providing blood samples for the primary endpoint. Participants with SARS-CoV-2 infection or antibody therapy (e.g. IVIG), between randomisation and providing blood samples will be excluded from the primary analysis. For safety reporting, participants will be included in the intervention group that they received, irrespective of the intervention they were randomised to.

The primary outcome will be the anti-spike IgG antibody against SARS-CoV-2, 21–35 days after completion of study vaccines, summarised as the GMC. Participants who miss a study blood draw, have SARS-COV-2 infection diagnosed by RAT, PCR or anti-nucleocapsid antibodies, or obtain an unscheduled dose of COVID-19 vaccine between the time of randomisation and the primary endpoint will be excluded from analysis. A Bayesian hierarchical two-part model will be used for the primary analysis as it is anticipated that a significant proportion of primary outcomes may be below the assay limit of detection.

Secondary endpoints described above will be analysed depending on the type of outcome; continuous immunological variables will be modelled with the two-part Bayesian model described; binary endpoints, including safety endpoints, will be analysed using a Bayesian logistic model; a Bayesian Poisson regression will be used to compare counts and rates. Further statistical detail is available in the statistical appendix which will be published separately.

### Interim analyses {21b}

The first analysis of the primary outcome will be performed after 260 eligible participants have completed 21–35 days of follow-up after receipt of their final study vaccine, and the results from the batched blood samples are available from the laboratory analysis. Subsequent analyses will be performed after every 100 additional participants have available laboratory results for the remainder of the trial until the maximum recruitment of 960 participants is reached.

### Methods for additional analyses (e.g. subgroup analyses) {20b}

Additional analyses may be conducted to examine for statistical interactions between anti-spike IgG serostatus at baseline, reported COVID-19 infection, number and type of vaccine doses received prior to randomisation, and type and intensity of immunosuppression received.

### Methods in analysis to handle protocol non-adherence and any statistical methods to handle missing data {20c}

No modifications or corrections to the endpoint will be made due to non-adherence to allocated intervention, in the context of a serious adverse event, or where non-adherence is due to site procedures or availability. In contrast, participants with missing data (e.g. blood draw) due to lost to follow-up or withdrawal from the study will be excluded from the analyses.

### Plans to give access to the full protocol, participant-level data and statistical code {31c}

The trial protocol and statistical code used for analyses will be made publicly available following publication of the primary results in the academic literature. Deidentified participant-level data may be made available upon reasonable request to the principal investigator after the publication of the results.

## Oversight and monitoring

### Composition of the coordinating centre and trial steering committee {5d}

The coordinating trial centre is located at the Department of Infectious Diseases at Alfred Hospital, in Melbourne. The Trial Steering Committee (TSC) is chaired by the Principal Investigator (PI) and includes representatives from the clinical, laboratory, statistical disciplines and consumer representatives. The TSC is responsible for the study conception, drafting, and completion of the study protocol and associated documents, recruitment plan, data monitoring and integrity, endpoint adjudication, and approving publications arising from the study.

### Composition of the data monitoring committee, its role and reporting structure {21a}

The Data Safety Monitoring Committee (DSMC) will consist of at least 3 members, and up to 6 members. The Chair will have expertise in clinical trial methods. Additional medical, and statistical experts will be selected to ensure the necessary expertise to oversee the trial, including an understanding of Bayesian adaptive clinical trials. The DSMC will meet at least three times per year to monitor and review accumulating safety reports and will make recommendations to the CPI on whether there are any ethical or safety reasons why the trial should not continue or should be modified prior to continuation. The DSMC will also review accumulating trial data for the primary analysis at pre-specified times detailed in the statistical appendix. The results of these analyses will be provided confidentially to the DSMC as an unblinded report, including recommendations to continue or stop recruitment into each stratum depending on whether the precision threshold has been met.

The Chair of the DSMC will be contacted for advice and independent review in the following situations: Following any SUSAR, a decision threshold is met, and any other situation where the Investigator feels independent advice or review is important. Full details of the DSMC’s function and workings are detailed in the trial DSMC charter.

### Adverse event reporting and harms {22}

This study will collect data on both solicited and unexpected adverse events (AE) following study intervention. At each contact with the participant, information will be sought on adverse events and details recorded in the eCRF. Solicited AEs are those events specifically captured in the diary card up to 7 days after each vaccination and for this study include pain, induration, erythema, temperature, headache, fatigue, chills, myalgia, joint pain, nausea, diarrhoea, chest pain.

Serious adverse event (SAE) is one which is deemed medically significant; warrants hospitalisation or emergency department presentation; results in congenital anomaly or permanent disability or incapacitation; and is life-threatening or causes death. SAEs are monitored continuously and have special reporting in the eCRF, and to the study sponsor and HREC within 24 h of an investigator becoming aware. All SAEs that occur from randomisation up to 28 days following the last dose of an investigational product will be documented and reported; SAEs that occur thereafter will only be documented and reported if they are deemed to be at least possibly related to the study vaccine. Suspected unexpected serious adverse reactions (SUSARs) are any SAE that is both suspected to be related to the trial treatment and is unexpected (i.e. not consistent with the available safety information in the investigator’s brochure (for unapproved products)/approved product information. A specific list of adverse events, and adverse events of special interest are defined and will be recorded if occurring throughout the course of the study for participants [39].

### Frequency and plans for auditing trial conduct {23}

Monitoring of study data at a site level to confirm compliance with study procedures from source notes will be coordinated by the study manager. For each site, the first monitoring visit occurs 1 month after enrolment has begun and every 6 months thereafter. In addition, the sponsor is responsible for providing an annual safety report to the administering HREC including a summary of the evolving safety profile of the trial.

### Plans for communicating important protocol amendments to relevant parties (e.g. trial participants, ethical committees) {25}

Protocol amendments will be approved by the HREC at the coordinating trial centre, and where necessary, local trial governance. Trial registration information at clinicaltrials.gov will be updated with protocol modifications.

### Dissemination plans {31a}

The results of BOOST-IC will be reported in a timely manner in the peer-reviewed scientific literature and at local and international conferences. All findings, whether negative or positive, will be reported. Lay summaries of study results will be made available to participants and distributed to the study consumer representative group.

## Discussion

BOOST-IC is a multi-centre, adaptive randomised clinical trial to assess the immunogenicity and safety of alternative booster regimens of COVID-19 vaccines in immunocompromised groups. This includes those living with HIV, solid organ transplantation, and certain haematological malignancies.

This study builds on local and international evidence that the study’s target populations are more likely to experience poorer outcomes following infection with SARS-CoV-2 and to have suboptimal immunological responses to current immunisation schedules. While the availability of COVID-19-targeted antivirals reduces the risk of severe infection, they are not licensed or proven to be effective for prevention. Administration of monoclonal antibodies, either alone or in combination, has previously proved useful in people unable to mount effective humoral responses. However, these are expensive, challenging to access, and their efficacy has been limited by the rapid evolution of SARS-CoV-2 variants characterised by their capacity to escape existing neutralisation responses. Hence, the development of endogenous immunological responses through vaccination remains an essential component of prevention strategies for COVID-19 in these groups.

Strengths of this study include its pragmatic design and its assessment of both immunological and safety outcomes. Immunological responses will be assessed objectively, while the blinding of participants and assessors to vaccine type and schedule will reduce contamination of responses. Additional strengths include the inclusion of a broad range of participants and levels of immunocompromise, and the design allows the inclusion of additional new vaccines as they become available.

Limitations of this study include the heterogeneous nature of enrolled participants with different immunosuppressive regimens, levels of hybrid immunity due to past infection and numbers and types of COVID-19 vaccines. Similarly, this study is conducted in the context of community transmission of COVID-19, and evolving COVID-19 policy which may influence trial participation, the desire by participants and their usual clinicians to obtain additional vaccines and therapies outside of the trial, which may affect results, introduce protocol deviations, or trial completion.

## Trial status

Recruitment commenced on December 17 2022 and will continue until December 2025. The current version of the protocol is version 7.0 (12 October 2023).

### Supplementary Information

Supplementary Material 1.

## Data Availability

Study results will be presented at conferences and via publications in biomedical journals relevant to infectious diseases and/or the clinical groups studied. Deidentified participant data may be made available upon request and submission of an appropriate research plan and associated ethical and institutional approvals.

## Abbreviations

AE: Adverse event
AEFI: Adverse events following immunisation
AESI: Adverse events of special interest
AIR: Australian Immunisation Register
ATAGI: Australian technical advisory group in immunisation
CAR-T: Chimeric antigen receptor-T cells
CD4: Cluster of differentiation 4
CD 20: Cluster of differentiation 20
ChAdOx1: Oxford–AstraZeneca COVID-19 vaccine
CLL: Chronic lymphocytic leukaemia
COVID-19: Coronavirus 2019
CRF: Case report form
CTMS: Clinical trial management system
DSMC: Data Safety Monitoring Committee
eCRF: Electronic case report form
GMC: Geometric mean concentration
HIV: Human immunodeficiency virus
HLA: Human leukocyte antigen
HREC: Human research ethics committee
ICH: Immunocompromised host
Ig: Immunoglobulin
IVIG: Intravenous immunoglobulin
MM: Multiple myeloma
mRNA: Messenger ribose nucleic acid
NHL: Non-Hodgkin lymphoma
PCR: Polymerase chain reaction
PI: Principal investigator
PLWH: People living with HIV
PRMS: Patient-reported measurement system
RAT: Rapid antigen test
SARS-CoV-2: Severe acute respiratory syndrome coronavirus 2
SAE: Serious adverse event
SOT: Solid organ transplantation
SUSAR: Suspected unexpected serious adverse reactions
TGA: Therapeutic Goods Administration
TSC: Trial Steering Committee
