# Supplementary material for: Bringing optimised COVID-19 vaccine schedules to immunocompromised populations (BOOST-IC): study protocol for an adaptive randomised controlled clinical trial
Source: Trials. 2024 Jul 17;25:485. doi: 10.1186/s13063-024-08315-2 (PMC11253462; doi:10.1186/s13063-024-08315-2)
Supplement: Supplementary file 1 — Supplementary Material 1. [file 13063_2024_8315_MOESM1_ESM.docx]

Appendix 1. BOOST-IC Protocol List of Sites

- Alfred Hospital, Melbourne, Victoria, Australia
- Monash Health, Clayton, Victoria, Australia
- University Hospital Geelong, Geelong, Victoria, Australia
- Austin Health, Heidelberg, Victoria, Australia
- St Vincent’s Hospital Melbourne, Victoria, Australia
- Royal Melbourne Hospital, Parkville, Victoria, Australia
- Peter MacCallum Cancer Centre, Parkville, Victoria, Australia
- St Vincent’s Hospital Sydney, New South Wales, Australia
- Westmead Hospital, Westmead, New South Wales, Australia
- Prince of Wales Hospital, Randwick, New South Wales, Australia
- Royal North Shore Hospital, St Leonards, New South Wales, Australia
- Royal Adelaide Hospital, Adelaide, South Australia, Australia
- Royal Brisbane and Women’s Hospital, Herston, Queensland, Australia
- Princess Alexandria Hospital, Woolloongabba, Queensland, Australia
- Melbourne Sexual Health Centre, Melbourne, Victoria, Australia
